# Health system financing paradigm in the state of São Paulo: a regional analysis

**DOI:** 10.11606/S1518-8787.2019053000796

**Published:** 2019-04-26

**Authors:** Adilson Soares

**Affiliations:** I Secretaria de Estado da Saúde. Programa de Pós-Graduação em Ciências da Coordenadoria de Controle de Doenças. São Paulo, SP, Brasil; II Secretaria de Estado da Saúde. Grupo de Apoio as Políticas de Prevenção e Proteção à Saúde. São Paulo, SP, Brasil

**Keywords:** Unified Health System, Health Care Rationing, Economics, Health Economics, Health Policy, Sistema Único de Saúde, Alocação de Recursos para a Atenção à Saúde, economia, Economia da Saúde, Política de Saúde

## Abstract

**OBJECTIVE:**

To analyze the allocation of financial resources in the Brazilian Unified Health System (SUS) in the state of São Paulo by level of care, health region, source of funds and level of government.

**METHODS:**

This is an exploratory study based on 2014 data extracted from the Public Health Budget Database, presented in absolute terms, relative terms and *per capita* .

**RESULTS:**

In 2014, R$52.1 bi were spent on public health, 58.0% having corresponded to the expenditures of the municipalities and 42.0% to those of the state government. Regional *per capita* spending varied from R$561.75 to R$824.85. As for the *per capita* spending on primary health care, which represented 37.5% of the municipalities’ total expenditure, the lowest value was found in the city of São Paulo and the highest, in Araçatuba. Campinas had the highest *per capita* expenditure on medium and high complexity care, while Presidente Prudente had the lowest. The highest regional percentage of the current net revenue spent on health was verified in Registro, and the lowest, in the city of São Paulo.

**CONCLUSIONS:**

The paradigm of the health sector’s financing in São Paulo revealed that the expenditure on primary health care, level elected by health policy as strategic because it depends on coordination and integral health care in the attention networks, was not considered a priority in relation to the expenditure with the medium and high complexity, exposing the iniquities in the state’s regions.

## RESUMO

**OBJETIVO:**

Analisar a alocação de recursos financeiros no Sistema Único de Saúde (SUS) no estado de São Paulo por nível de atenção, região de saúde, fonte de recursos e ente federado.

**MÉTODOS:**

Trata-se de estudo exploratório circunscrito ao exercício de 2014. Os dados extraídos do Sistema de Informações sobre Orçamentos Públicos em Saúde estão apresentados em valores absolutos, relativos e *per capita* .

**RESULTADOS:**

Em 2014 observou-se um gasto público com saúde de R$52,1 bi, sendo 58,0% relativos ao gasto dos municípios e 42,0% relativos ao gasto do governo do estado. O gasto regional *per capita* variou de R$561,75 a R$824,85. Já o gasto *per capita* com atenção primária à saúde, que representou 37,5% do gasto total dos municípios, foi menor na região da Grande São Paulo e maior em Araçatuba. A região de Campinas apresentou o maior gasto *per capita* com atenção de média e alta complexidade, enquanto Presidente Prudente teve o menor. O maior percentual regional da receita corrente líquida gasto com saúde foi verificado em Registro, e o menor na Grande São Paulo.

**CONCLUSÕES:**

O paradigma de financiamento do setor da saúde em São Paulo revelou que o gasto com a atenção primária, nível eleito pela política de saúde como estratégico porque dele dependem a coordenação e o cuidado integral à saúde nas redes de atenção, não recebeu prioridade em relação ao gasto com a média e a alta complexidade, expondo as iniquidades nas regiões do estado.

## INTRODUCTION

The Brazilian Unified Health System (SUS), defined as a right of all and a duty of the State, must be guaranteed through economic and social policies ^1^ . With the intent of correcting a profound social deficit, accumulated over decades, the text of the Constitution established social security in Brazil and showed how the health sector’s development depends intrinsically on social and economic policies. In this way, when analyzing the health sector, Soares ^2^ states that it is not “possible to treat health policy issues in isolation, and without considering their articulation and reliance on economic issues, because the level of investments to be made in this policy, for instance, derives from them” (p. 31). The definitions about the health sector’s financing, which do not appear in the Federal Constitution of 1988 ^1^ and in the Organic Laws of Health ^3^ ^,^ ^4^ , were postponed for future discussions, which occurred belatedly and were mainly materialized in Constitutional Amendment 29/2000 ^5^ (EC 29), Supplementary Law 141/2012 ^6^ , Constitutional Amendment 86/2015 ^7^ and Constitutional Amendment 95/2016 ^8^ , among others of SUS’s legal and regulatory framework references.

The articulation between health policy and economic policy was not enough to increase federal government spending on health care during Brazil’s period of economic growth. According to Soares ^2^ , the federal public sector’s spending in the period from 1995 to 2012 remained practically constant, rotating around 1.7% of the gross domestic product (PIB), and decreasing its share percentage in relation to the Union’s general budget (OGU), the current gross revenue (RCB) and the current net revenue (RCL). This policy of containment of public spending on health contributed to the federal government to increase the primary surplus, which grew 659.0% in the period, to ensure the payment of the interest on the domestic debt, which grew 264.0% in the period.

The economic results sought for and obtained since the mid-1990s are aligned with the political and economic project that brought to Brazil, among other things, the idea of the centrality of the fiscal policy with priority given to the realization of primary surpluses, having as consequence the financialization of public budgets ^2^ .

This policy, despite the constitutionalization of the right to health, maintained the historical underfunding of the public health sector in the country, and deepened the funding crisis by allowing the inflection of the levels of government share in the sector’s spending at the expense of subnational levels of government, states and municipalities, which have lower tax collection capacity. The transition of public health funding in Brazil, which becomes clearer in the 1990s, reveals a movement toward a greater relative share of the states and, mainly, of the municipalities in the total expenses. The federal government’s spending on health care, which accounted for 74.4% of the total expenses in Brazil in 1990, started representing only 45.5% in 2012, while the states’ spending rose from 13.5% in 1990 to 26.8% in 2012, and the municipalities’ spending rose from 12.1% to 28.2% in the same period ^2^ .

This transition in spending was accompanied by the transition in the obligations of execution of public health actions and services (PHAS), with greater responsibilities assigned to the states and municipalities. It reveals the inequity in the system’s financing, as these governments start spending a significant and ever-increasing share of their budgets on the health sector, mainly the municipalities – for which the limit of spending of their RCL on PHAS ^6^ is 15% ^6^ , and which spent, on average, 22.9% in 2014 ^9^ .

The foundations of the Brazilian health system are rooted in the recommendations outlined in the Dawson Report ^10^ of the beginning of the 20th century and in the Alma-Ata Declaration ^11^ of the end of the 1970s which, among other things, identify in primary health care (PHC) the power to coordinate and organize the health care network ^12^ . PHC has the important role of organizing and rationalizing the resources of the entire system ^13^ . The consolidation of this model – which aims to, among other things, restructure the health system’s financing – gained momentum mainly since the editing of the 1993 Basic Operating Standard (NOB 93), which triggered the process of regular and automatic transfer of the federal government’s resources to municipalities and states, expediting the process of municipal investments at this level of care ^14^ .

The discussions developed under the scope of SUS’s inter-manager committees, which culminated in the publication of the Health Compact ^15^ and of the National Policy of Basic Attention ^16^ in 2006, were central in the development and definition of priorities and resources for PHC policies in Brazil. The search for equity in health, with the aim of complying with SUS’s regulatory and legal framework, includes, among other things, the analysis of the sector’s financing.

Thus, the objective of this study was to analyze the allocation of resources in the Brazilian Unified Health System in the state of São Paulo and, from the perspective of the investments made by level of care, health region, source of funds and level of government, discuss the institution and the instituted in public health policy.

## METHODS

This is an exploratory study based on the collection and analysis of 2014 data on the implementation of the health sector budget in the municipalities and government of the state of São Paulo. The Public Health Budget Database (SIOPS), which allows the institutional estimation and dissemination of health spending data, was used as main basis for the collection of secondary information.

The budgetary execution data were extracted of the Budgetary Execution Summary Report (RREO) do SIOPS, organized and presented *per capita* and in absolute and relative terms. Priority was given to the study of comparisons between the investments in PHC actions, medium and high complexity (MHC) actions and health surveillance actions, without disregarding the expenses on other budgetary sub-functions.

As public health policies in Brazil use the term “basic health care” (BHC) and not “PHC”, we adopted the theoretical framework of Mello et al. ^17^ , who describe “basic care”, “primary care” and “primary health care” as “concepts that are indistinguishable from each other” (p. 210). To facilitate the differentiation between the levels of care, the term “MHC” was used to refer to the budgetary sub-function “hospital and outpatient care”, the term “health surveillance” to refer to the total expenditure on the associated sub-functions of “health surveillance” and “epidemiological surveillance”, and the term “pharmaceutical services” to refer to the budgetary sub-function “prophylactic and therapeutic support.”

This decision was made according to the methodology used by Brazil in its chart of accounts of public finances and in the satellite health accounts that make up the National Accounts System, which defines the budgetary sub-functions related to the levels of care, treating the system’s financing in blocks and also in relation to these sub-functions. In the health sector’s case, these sub-functions are defined in the financial and budgetary management system as: basic health care, hospital and outpatient care, sanitary surveillance, epidemiological surveillance, prophylactic and therapeutic support, feeding and nutrition and other sub-functions.

For comparison and analysis of the data, they were adjusted by eliminating the population effect to correct the gross value, resulting in *per capita* values. The population data were extracted from the database of the Brazilian Institute of Geography and Statistics (IBGE). For the analysis of the population coverage of PHC and MHC, the number of individuals registered in the Ministry of Health’s Primary Health Care Database (SIAB/MS) ^18^ and the number of users of the supplementary health care services, extracted from the database of the Ministry of Health’s Supplementary Health Agency (ANS/MS), were considered ^19^ .

As health policies, planning and programming in the state of São Paulo are carried out by a coordinated regional structure composed of 17 regional health departments, the analysis of the data sought to establish comparisons between them.

## RESULTS

Initially, it is important to highlight that the appropriation of health spending on inadequate sub-functions was noted. Of the 645 municipalities in São Paulo, 18 allocated resource expenditures relating to PHC actions in the MHC sub-function, and five allocated the PHC sub-function’s resources under the heading “other sub-functions.” The inadequate allocation of expenditure on MHC by 31.8% of the total number of municipalities and of expenditure on health surveillance actions by 21.2% of them was also found. These inadequacies justify the significant percentage of resources allocated under the heading “other sub-functions.” It was also found that the state government allocated the spending on the payroll of the human resources who develop health surveillance actions in the MHC sub-function.

The state of São Paulo spent R$51.8 billion on PHAS in 2014, of which R$29.9 billion (58.0%) corresponded to the municipalities’ spending and R$21.9 billion (42.0%) to the state government’s ( Table 1 ). Of this total, 22.1% was spent on PHC, 58.1% on MHC, 4.9% on pharmaceutical services, 1.4% on health surveillance, 0.01% on food and nutrition, and 13.4% on other sub-functions, the expense *per capita* being R$277.77, R$729.99, R$61.49, R$17.71, R$0.15 and R$168.40, respectively, for these levels of care and sub-functions. The municipalities assumed 96.3% of the total spending on PHC in 2014. The total *per capita* spending on PHAS in the state of São Paulo in 2014 was R$1,255.52.

**Table 1:** Total health spending by level of care and level of government in the state of São Paulo, 2014.

Categories	PHC	MHC	Pharmaceutical Services	Health Surveillance	Food and nutrition	Other sub-functions	Total
Health spending by the municipalities	11,036.04	12,191.83	614.47	617.10	6.04	5,435.77	29,901.25
% spending by sub-function	36.9%	40.7%	2.1%	2.1%	-	18.2%	100.0%
Spending *per capita*	267.45	295.46	14.89	14.96	15.00	131.73	724.63
% private resources spent on health							23.8%
Health spending by the state manager	425.99	17,930.79	1,922.96	113.62	0.00	1,512.94	21,906.29
% spending by sub-function	1.9%	81.9%	8.8%	0.5%	0.0%	6.9%	100.0%
Spending *per capita*	10.32	434.54	46.60	2.75	0.00	36.66	530.88
% own resources spent on health							12.5%
Total health spending in the state of São Paulo	11,462.03	30,122.62	2,537.43	730.72	6.04	6,948.71	51,807.54
% spending by sub-function	22.1%	58.2%	4.9%	1.4%	-	13.4%	100.0%
Spending *per capita*	277.77	729.99	61.49	17.71	0.15	168.40	1,255.52

Source: Prepared by the author based on data of the Ministry of Health – Public Health Budget Database, the National Health Fund, and the Brazilian Institute of Geography and Statistics.

Notes:

1. PHC: primary health care; MHC: medium and high complexity

2. Considering the population’s 41,263,969 inhabitants.

3. Values in millions of reais at current prices.

By analyzing the data according to health region ( Table 2 ), it is possible to note that the total regional *per capita* spending ranged from R$597.71 to R$896.11, Franca being the region with the lowest spending, and Campinas the one with the highest. In relation to PHC, three regions showed *per capita* expenditures below the state average, the city of São Paulo being the one that invested the least in it (R$212.51). Of the regions that invested above the state average, the highlight is the region of Araçatuba (R$490.72), followed by the regions of Presidente Prudente, São José do Rio Preto, Registro, Marília, Barretos, Bauru, Ribeirão Preto, Franca, Taubaté, Araraquara, Santos and Sorocaba.

**Table 2:** Municipal spending on health by level of care and sub-function and health region of the state of São Paulo, 2014.

Region	PHC	MHC	Health surveillance	Pharmaceutical services	Other sub-functions	Total	Coverage PHC	Coverage MHC
Araçatuba	490.72	177.59	30.31	11.19	15.56	725.37	64.94	75.00
Araraquara	286.78	275.83	22.06	7.78	135.79	728.25	34.85	62.11
Baixada Santista	279.78	450.25	29.43	10.47	80.82	850.75	30.62	59.14
Barretos	372.32	354.73	24.11	8.96	2.37	762.49	39.46	68.58
Bauru	371.47	128.94	18.03	14.37	113.80	646.61	36.26	74.67
Campinas	220.02	468.92	16.98	25.45	164.73	896.11	26.85	51.80%
Franca	341.43	231.41	12.56	10.31	1.99	597.71	33.09	66.19
São Paulo	212.51	282.77	11.07	15.54	169.05	690.94	36.18	50.17
Marília	388.75	240.75	23.29	19.14	54.15	726.07	58.58	78.93
Piracicaba	246.47	273.89	14.08	9.58	197.53	741.55	31.02	54.11
Presidente Prudente	466.54	114.61	17.26	19.26	36.21	653.88	61.28	76.74
Registro	446.91	249.67	13.47	9.45	39.56	759.07	64.88	90.17
Ribeirão Preto	370.18	266.02	36.14	13.51	31.59	717.44	30.94	60.06
São João da Boa Vista	258.72	382.67	16.79	2.23	55.21	715.62	46.85	65.18
São José do Rio Preto	452.84	127.71	17.27	15.24	104.14	717.20	42.88	67.36
Sorocaba	262.61	353.04	8.41	11.88	56.54	692.47	25.26	68.61
Taubaté	324.37	297.69	11.53	9.01	111.89	754.49	33.51	64.80

Mean of the state	250.62	276.87	14.01	13.95	123.58	724.63	36.02	57.40

Source: Prepared by the author based on data of the Ministry of Health – Public Health Budget Database, the National Health Fund, and the Brazilian Institute of Geography and Statistics.

PHC: primary health care; MHC: medium and high complexity

Notes:

1. *Per capita* values in reais at current prices.

2. Values relating to expenses exclusive to the municipal agency.

3. “Other sub-functions” includes “administrative sub-functions” and “food and nutrition”.

4. The PHC coverage is based on the number of individuals registered in the Family Health Strategy and the MHC coverage, on the number of individuals using supplementary health services.

In relation to MHC, the region that invested the least in it was Presidente Prudente (R$114.61), followed by nine other regions with investments below the state average. Seven regions invested in MHC above the state average, starting with Campinas (R$468.92), followed by Baixada Santista, São João da Boa Vista, Barretos, Sorocaba, Taubaté and the city of São Paulo.

Ribeirão Preto was the region that most invested in health surveillance (R$36.14), and Sorocaba was the region that invested the least in it (R$8.41). In relation to pharmaceutical services, Campinas was the region that spent the most in them (R$25.45), while São João da Boa Vista was the one that spent the least (R$2.23).

The municipalities’ average *per capita* expenditure on other sub-functions was R$123.58, having in the region of Franca the lowest expenditure ($1.99), and Piracicaba having spent the most (R$197.53).

The public health care coverage in the state of São Paulo is 36.0% for PHC actions and 57.4% for MHC actions. According to these data, the state of São Paulo is the one with the lowest health care coverage in Brazil, except for primary care in the state of Amapá ^18^ ^,^ ^19^ .

The region with the lowest PHC coverage is Sorocaba (25.3%), which however has a 68.6% MHC coverage. The second region with the lowest PHC coverage is Campinas (26.8%), having also corresponded to the second lowest MHC coverage in the state (51.8%). The third region with lowest PHC coverage is Baixada Santista (30.6%), its MHC coverage being 59.1%. Then comes the region of Ribeirão Preto, with 30.9% PHC coverage and 60.1% MHC coverage. The greatest PHC coverages were verified in Registro and Araçatuba, with 64.9%, being respectively the first (90.2%) and the fourth (75.0%) greatest MHC coverages; followed by Presidente Prudente, with 61.3% PHC coverage, which also corresponded to the third greatest MHC coverage (76.7%).

The relative regional share in municipal spending on PHCS is presented in Table 3 , using as markers the regions with highest spending. The data demonstrate heterogeneity in spending, with the regions of Araçatuba, Campinas, Ribeirão Preto, Campinas and Piracicaba having invested the most in PHC, MHC, health surveillance, pharmaceutical services and other sub-functions, respectively. The ones that invested the least were the city of São Paulo, which represents 43.3% of the spending on PHC in the region of Araçatuba; Presidente Prudente, which represents 24.4% of the spending on MHC in the region of Campinas; Sorocaba, which represents 23.3% of the spending on health surveillance in the region of Ribeirão Preto; São João da Boa Vista, which represents 8.8% of the spending on pharmaceutical services in the region of Campinas; and Franca, which represents 1.0% of the spending on other sub-functions in the region of Piracicaba.

**Table 3:** Relative regional share of municipal health spending in the state of São Paulo, using as markers the regions with highest spending by level of care and sub-function, 2014.

Region	PHC	MHC	Health surveillance	Pharmaceutical services	Other sub-functions	Total
					
	Araçatuba = 100.0%	Campinas = 100.0%	Ribeirão Preto = 100.0%	Campinas = 100.0%	Piracicaba = 100.0%	Campinas = 100.0%
Araçatuba	100.0	37.9	83.9	44.0	37.9	80.9
Araraquara	58.4	58.8	61.1	30.6	68.7	81.3
Baixada Santista	57.0	96.0	81.4	41.1	40.9	94.9
Barretos	75.9	75.6	66.7	35.2	1.2	85.1
Bauru	75.7	27.5	49.9	56.5	57.6	72.2
Campinas	44.8	100.0	47.0	100.0	83.4	100.0
Franca	69.6	49.3	34.8	40.5	1.0	66.7
São Paulo	43.3	60.3	30.6	61.1	85.6	77.1
Marília	79.2	51.3	64.4	75.2	27.4	81.0
Piracicaba	50.2	58.4	39.0	37.6	100.0	82.8
Presidente Prudente	95.1	24.4	47.8	75.7	18.3	73.0
Registro	91.1	53.2	37.3	37.1	20.0	84.7
Ribeirão Preto	75.4	56.7	100.0	53.1	16.0	80.1
São João da Boa Vista	52.7	81.6	46.5	8.8	27.9	79.9
São José do Rio Preto	92.3	27.2	47.8	59.9	52.7	80.0
Sorocaba	53.5	75.3	23.3	46.7	28.6	77.3
Taubaté	66.1	63.5	31.9	35.4	56.6	84.2

Source: Prepared by the author based on data of the Ministry of Health – Public Health Budget Database, the National Health Fund, and the Brazilian Institute of Geography and Statistics.

PHC: primary health care; MHC: medium and high complexity

Note: Values exclusively related to the expenditure of municipalities.

Considering that “Primary Health Care is characterized by a set of health actions, within individual and collective contexts, that involve the promotion and protection of health, the prevention of diseases, diagnosis, treatment, rehabilitation, harm reduction and the maintenance of health [...]” ^20^ (p. 3), beyond the recommendations of the Dawson ^10^ and Alma-Ata ^11^ Reports that preventive, protective and promotive actions should be part of the first level of health care, in this study, PHC data (including the values associated with the sub-functions of health surveillance, pharmaceutical services and food and nutrition) were compared to MHC data ( Figure ). In this more comprehensive analysis, it is found that there is no significant change in PHC spending in relation to the regional comparative data in Table 3 when using its expanded concept, and that the iniquities in the spending on these levels of care in the state of São Paulo’s health regions persist.

**Figure:**
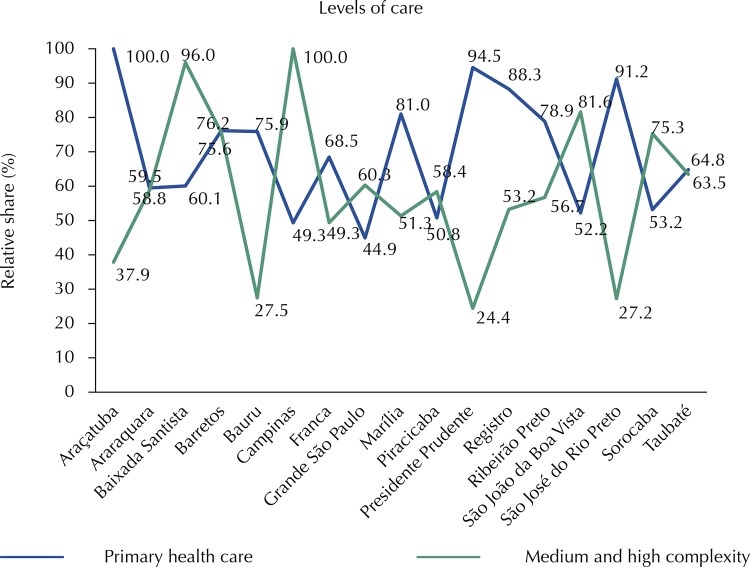
Relative regional share of municipal health spending by level of care in the state of São Paulo, using the concept of primary health care, 2014.

Source: Prepared by the author based on data of the Ministry of Health – Public Health Budget Database, the National Health Fund, and the Brazilian Institute of Geography and Statistics.

In relation to the sources of funding that supported the health expenditures of the state of São Paulo’s municipalities in 2014 ( Table 4 ), the investments made with own resources totaled R$21.6 billion, which corresponds to 72.1% of all investment on PHAS in the municipalities, while the intergovernmental transfers carried out by the federal and state government to the municipalities totaled R$7.8 billion, and the heading “other sources”, R$721 million. Together, these two sources corresponded to 27.9% of the total invested. It should be noted that in 2014, in relation to 2012, the relative share of the federal government’s total spending on health care fell from 40.5% to 37.4%, and that the share of PHC in the total federal funds transferred to the states and municipalities was reduced from 34.0% to 31.0%.

**Table 4:** Municipal health spending by source of funds and health region of the state of São Paulo, 2014.

Region	Municipal resources	State and federal resources	Other sources	Total	% municipal resources/total spending	% municipal resources invested in Health	Per capita RITCL
Araçatuba	369.59	146.88	1.47	517.94	71.4	26.2	1,846.44
Araraquara	448.33	213.37	0.06	661.76	67.7	28.4	1,604.98
Baixada Santista	960.20	438.37	17.08	1,415.66	67.8	22.6	2,385.65
Barretos	195.30	115.77	0.99	312.06	62.6	24.8	1,825.80
Bauru	762.31	282.63	5.27	1,050.21	72.6	26.3	1,671.71
Campinas	2,771.08	832.20	6.57	3,609.85	76.8	27.0	2,346.31
Franca	287.97	98.58	1.84	388.39	74.1	27.0	1,541.64
São Paulo	10,004.19	3,207.72	288.62	13,500.53	74.1	21.3	2,246.29
Marília	486.57	278.64	10.54	775.75	62.7	25.7	1,684.74
Piracicaba	692.96	352.30	0.88	1,046.14	66.2	25.4	1,803.49
Presidente Prudente	326.35	144.38	1.03	471.76	69.2	24.3	1,766.15
Registro	154.50	47.17	5.99	207.66	74.4	33.2	1,639.09
Ribeirão Preto	679.40	270.67	127.06	1,077.13	63.1	26.7	1,772.45
São João da Boa Vista	391.38	157.53	0.63	549.54	71.2	29.6	1,623.88
São José do Rio Preto	743.37	306.08	1.69	1,051.14	70.7	25.0	1,897.16
Sorocaba	1,119.88	419.65	13.44	1,552.97	72.1	28.1	1,655.85
Taubaté	1,245.38	439.87	27.51	1,712.75	72.7	27.8	1,846.18

Total	21,638.76	7,751.81	510.68	29,901.25	72.4	23.8	2,067.34

Source: Prepared by the author based on data of the Ministry of Health – Public Health Budget Database, the National Health Fund, and the Brazilian Institute of Geography and Statistics.

RITCL: tax revenue and constitutional and legal transfers

Note: Values in millions of reais at current prices in 2014.

The region of Campinas was the one that most invested own resources in health in relation to the region’s total expenditure (76.8%), followed by Registro (74.4%) and Franca (74.1%). The lowest relative shares in the total spending on health were found in the regions of Barretos (62.6%), Ribeirão Preto (63.1%) and Marília (62.7%).

These percentages indicate that in the state of São Paulo, the municipalities’ shares in the maintenance of PHCS correspond to values that are mostly higher than those of other levels of government. The municipal investments directly impacted the percentage of RCL resources – own resources of the municipality – spent on health, 23.8% on average, above the minimum 15% percentage set by EC^29^. As for the state of São Paulo’s government, it spent 12.5% of its RCL on PHCS ( Table 1 ), more than the minimum 12% percentage set by EC^29^, however close to it.

The region of Campinas, the one that most invested in health (R$896.11 *per capita* ), was the seventh with greater application of own resources in the sector (27.0%) and had the second greatest investment capacity in relation to its tax revenue and legal constitutional transfers (RITCL), with R$2,346.31 *per capita* . The second region that most invested was Baixada Santista, with R$850.75 *per capita* , being the sixteenth (penultimate) in the application of own resources in health (22.6%) and had the greatest investment capacity in relation to the RITCL (R$2,385.65 *per capita* ). The third region that most invested was Barretos, with R$762.49 *per capita* , being the fourteenth in spending with own resources (22.6%) and had the seventh greatest investment capacity in relation to the RITCL (R$2,385.65 *per capita* ). The region that least invested in health was Franca, with R$597.71 *per capita* , being the sixth that least invested its own resources in health (27.0%) and had the smallest investment capacity in relation to the RITCL (R$1,541.65 *per capita* ). The second region that least invested in health was Bauru, with R$646.61 *per capita* , being the ninth region that most invested its own resources in health (26.3%) and had the twelfth smallest investment capacity in relation the RITCL (R$1,671.71 *per capita* ). The third region that least invested in health was Presidente Prudente, with R$653.88 *per capita* , being the fifteenth in investment of own resources (24.3%) and had the tenth smallest investment capacity in relation to the RITCL (R$1,766.15).

## DISCUSSION

The paradigm of the health sector’s financing in the state of São Paulo revealed the protagonism of municipalities in the total investments in public health actions and services. Primary health care, a level elected by public health policy in Brazil as strategic because it depends on coordination and integral health care in care networks, received priority in the application of resources in some regions of the state; however, as well as in the level of attention of medium and high complexity, this occurred unevenly and with low coverage.

The level of investment made by the municipalities was very close to the limit set by the regulatory and legal framework of the field of health. Based on this, great difficulty of expansion for the resolution of SUS’s central problems in the state, low coverage and correction of regional inequities, are expected.

These problems emerged and have been worsening, internally, due to the limited assistance from the state government for the increase of the spending on PHC, as it focuses its investments on MHC instead, and externally, due to the impossibility of expansion of federal resources, because the federal government has been reducing its relative share in the total spending on health and the relative share of primary care resources, in what concerns fund to fund transfers, in the total expenses.

The health system in Brazil, which has faced chronic underfunding since its constitutionalization, is threatened by the sector’s lack financing due to the implementation of an austere economic policy, that presides over social policies. This policy is centered on fiscal adjustment with containment of primary spendings ^21^ in the twenty years of validity of Constitutional Amendment 95/2016 (from 2017 to 2036), which, according to studies, should cause the health sector to lose R$415 billion for the financing of PHAS ^22^ . In this way, the maintenance of the downward trend in the relative participation of the Union in the total spending on PHCS is expected, as well as reduction of PHC investments proportionally to the other levels of care, and maintenance of regional inequities in spending.

In this scenario, this study reveals the unsustainability of the state of São Paulo’s health care policy in the next few years, showing that the share of the state’s municipalities in PHC spending is 96.3%, even at the limit of the municipal RCL’s average capacity of investments in health (23.8%).

The allocation of spending in inappropriate headings may lead to bias in the analysis of the data, representing a limitation of the study. Based on this, policymakers should qualify the information entered in SIOPS, seeing as this system is a source of information for the development of public health policies in Brazil, and the basis for national and international studies comparing investments in the field of health between levels of government and countries.

The analysis undertaken in this study leads to the consideration that the reformulation of the health care model established in the state of São Paulo’s public policies must be subjected to the actual implementation of regionalization, with the consequent clear definition of the levels of government responsibility for health services and actions, and the redistribution of financing to achieve greater fairness in the allocation of resources and in the access of the population to the actions and services at all levels of care.

One of the achievements of the field of health and teaching of the Brazilian health reform movement, embodied in the constitutional text, is that health is realized via economic and social policies; thus, the solutions to current health dilemmas in Brazil and the reformulations proposed cannot, should not be and are not restricted to the sector. In this way, the discussion on SUS’s financing must be carried out in the context of social security, of the reassessment of the federal pact and of the tax reform.

Finally, it is important that studies articulating and expanding the scope of this research to the national level are conducted, contributing to the discussion and correction of systemic inequities, and supporting the health care model with focus on primary health care as the level responsible for the managing and coordinating of the health care network in Brazil.
